# Serie de casos: tuberculosis extremadamente resistente a drogas en Colombia, 2006-2016

**DOI:** 10.7705/biomedica.4842

**Published:** 2019-12-30

**Authors:** Angie Zabaleta, Claudia Llerena

**Affiliations:** 1 Grupo de Micobacterias, Dirección Redes en Salud Pública, Instituto Nacional de Salud, Bogotá, D.C., Colombia Grupo de Micobacterias Dirección Redes en Salud Pública Instituto Nacional de Salud BogotáD.C Colombia

**Keywords:** Mycobacterium tuberculosis, tuberculosis extremadamente resistente a drogas, tuberculosis resistente a múltiples medicamentos, tuberculosis pulmonar, Colombia, Mycobacterium tuberculosis, extensively drug-resistant tuberculosis, tuberculosis, pulmonary, tuberculosis multidrug-resistant, Colombia

## Abstract

**Introducción.:**

La tuberculosis extremadamente resistente a los medicamentos (TB-XDR) es el resultado de deficiencias en la administración del tratamiento y en la prevención de la transmisión de la enfermedad; además, es un reto para los programas nacionales de control de la tuberculosis.

**Objetivo.:**

Describir las características clínicas y epidemiológicas de los casos de TB-XDR diagnosticados en Colombia.

**Materiales y métodos.:**

Se trata de un estudio de serie de casos, de pacientes con diagnóstico de TB-XDR, de 2006 a 2016 en Colombia. Las fuentes de información fueron el formato único de vigilancia y la base de datos del Laboratorio Nacional de Referencia. Se analizaron las variables: entidad territorial de procedencia, sexo, edad, régimen de afiliación, forma de tuberculosis, coinfección tuberculosis-HIV, patrón de sensibilidad a los fármacos de segunda línea y resultado (vivo o muerto).

**Resultados.:**

En el período de estudio, se diagnosticaron 51 casos de TB-XDR, 28 hombres y 23 mujeres, con un promedio anual de cinco casos. En los departamentos de Antioquia, Valle del Cauca y Atlántico, se presentaron 46 (90 %) de los casos. El rango de edad fue de 5 a 81 años y, la mediana, de 40 años. De los 51 pacientes, 32 (63 %) estaban afiliados al régimen subsidiado de salud y 46 (90 %) presentaron tuberculosis pulmonar; de los 22 a quienes se les practicó la prueba para HIV, en tres (13,6 %) había coinfección con HIV, y 29 (57 %) murieron. Los fármacos a los cuales hubo mayor resistencia, fueron ofloxacina en 45 (88 %) casos y amikacina en 43 (84 %).

**Conclusión.:**

La TB-XDR se presentó principalmente en formas pulmonares, lo cual aumenta la probabilidad de transmisión en la comunidad y se refleja en la aparición de tuberculosis resistente en menores de 15 años. La población más afectada es la económicamente activa, con una gran mortalidad. El Programa Nacional de Prevención y Control de la Tuberculosis debe generar estrategias para evitar la propagación de cepas resistentes.

## Abstract (en)

**Introduction:**

Extensively drug-resistant tuberculosis (XDR-TB) is a result of deficiencies in the administration of treatment and transmission of the disease, being a challenge for national programs of tuberculosis control.

**Objective:**

To describe the clinical and epidemiological characteristics of the cases diagnosed in Colombia.

**Materials and methods:**

Case series study, performed on people diagnosed with XDRTB during 2006 to 2016 in Colombia. Information source: unique surveillance format and database of the national reference laboratory. The variables territorial entity of origin, gender, age, affiliation regime, form of tuberculosis, tuberculosis-HIV coinfection, sensitivity pattern to second line drugs and live/dead outcome were analyzed.

**Results:**

In this period, 51 XDR-TB cases have been diagnosed, with an annual average of five cases. Antioquia, Valle del Cauca and Atlántico present 90% (46) of cases. Age range: 5 to 81 years, median: 40 years. There were 28 men (55%) of the cases. Subsidized affiliation regime: 63% (32/51). Pulmonary form: 90% (46/51). TB-HIV coinfection: 13.6% (3/22). The drugs with greater resistance: ofloxacin 90% (45/51) and amikacin 86% (43/51). Dead unlink: 57% (29/51).

**Conclusion:**

XDR-TB occurred mainly in pulmonary forms increasing the probability of transmission in the community, which is reflected in the appearance of resistance in children under 15 years of age. The most affected population is economically active, with high mortality. The national program of tuberculosis control must generate strategies to avoid the spread of resistance that is affecting children and the economically active population, with high mortality.

La tuberculosis resistente a múltiples medicamentos (TB-MDR) es la producida por *Mycobacterium tuberculosis* resistente a los fármacos isoniacida y rifampicina, con resistencia a otros fármacos de primera línea o sin ella. La tuberculosis extremadamente resistente a medicamentos (TB-XDR) es la producida por *M. tuberculosis* resistente a la isoniacida y la rifampicina, y, al menos, a una fluoroquinolona y a un medicamento inyectable de segunda línea, como kanamicina, amikacina o capreomicina.

La resistencia se puede generar por deficiencias en el tratamiento de la enfermedad (adquirida) o por la transmisión de cepas resistentes en la comunidad (primaria); es una amenaza para lograr el éxito del tratamiento y, por consiguiente, el control de la enfermedad. Además, la infección concomitante con HIV u otras enfermedades que debilitan el sistema inmunológico, aumenta la probabilidad de que las personas infectadas desarrollen la enfermedad y tengan un mayor riesgo de muerte ^1^^-^^4^.

Los dos factores de riesgo más asociados con la aparición de TBXDR, son el fracaso del tratamiento en pacientes que están recibiendo medicamentos como quinolonas y aminoglucósidos, y el contacto estrecho de una persona con enfermos de tuberculosis con este tipo de resistencia o que estén presentando falla terapéutica con un esquema de segunda línea ^1^^,^^3^.

En 2016, se notificaron 6,3 millones de casos nuevos de tuberculosis en el mundo, de los cuales 153.119 fueron de TB-MDR y resistentes a rifampicina, y 8.014 de TB-XDR. Europa aportó el mayor número de casos, con 3.114. En las Américas, 221.008 fueron casos nuevos, de los cuales, 3.715 fueron de TB-MDR/RR y 112 de TB-XDR. En Colombia, hubo 12.439 casos nuevos, de los cuales, 150 fueron de TB-MDR/RR y cinco de TB-XDR ^2^^,^^5^.

En el mundo, para el 2016, se notificaron 8.014 casos de TB-XDR; sin embargo, es de resaltar que puede existir un subregistro debido a que, de los casos notificados como TB-MDR/RR, solo el 39 % accedió a pruebas de sensibilidad a los fármacos de segunda línea (quinolonas e inyectables) ^2^.

Llerena, *et al*., publicaron el estudio ‘Prevalencia de la resistencia de *Mycobacterium tuberculosis* a quinolonas y fármacos inyectables en Colombia, 2012-2013’, en el cual analizaron 489 aislamientos de cepas resistentes a la isoniacida, la rifampicina o ambas; 438 no tenían antecedentes de tratamiento antituberculoso con fármacos de segunda línea y, entre ellos, no se encontraron casos de TB-XDR; 51 casos registraban haber recibido previamente estos medicamentos, de los cuales, 7 (13,7 %) presentaron TB-XDR ^6^.

El objetivo del presente trabajo fue describir las características clínicas y epidemiológicas de los casos de TB-XDR diagnosticados en Colombia durante el período de 2006 a 2016.

## Materiales y métodos

Se trata de una serie de casos, estudio llevado a cabo en pacientes con diagnóstico de TB-XDR durante un periodo de diez años en Colombia.

Las fuentes de información fueron el formato único de vigilancia de las micobacterias, el cual se recibe en el Laboratorio Nacional de Referencia y su base de datos de farmacorresistencia.

La metodología utilizada por la Red Nacional de Laboratorios para determinar la sensibilidad a los fármacos antituberculosos kanamicina, amikacina, capreomicina, ofloxacina, levofloxacina, moxifloxacina y etionamida, incluyó: proporciones múltiples en medio de Löwenstein-Jensen en los cultivos que se procesaron hasta el año 2012, y en el sistema BACTEC MGIT™, en los recibidos después de este año, cuando se implementó esta técnica en el dicho Laboratorio ^7^.

Se analizaron las variables: entidad territorial de procedencia, sexo, edad, régimen de afiliación, forma de tuberculosis, coinfección tuberculosis-HIV, patrón de sensibilidad a los fármacos de segunda línea y resultado (vivo o muerto).

Se calcularon las medidas de tendencia central para las variables numéricas y los porcentajes para las categóricas. Se analizó el porcentaje de resistencia a los fármacos de segunda línea (kanamicina, amikacina, capreomicina, ofloxacina, levofloxacina, moxifloxacina y etionamida). Los datos se organizaron, tabularon y analizaron mediante las herramientas Excel™ y Epidat 3.1™.

### Aspectos éticos

Según la Declaración de Helsinki de 2013, el Council for International Organizations of Medical Sciences (CIOMS) y la Resolución 8430 de 1993 del Ministerio de Salud, esta es una investigación sin riesgo, debido a que se trabajó con fuentes secundarias de información y no se hicieron intervenciones en las personas.

## Resultados

En los diez años analizados, se identificaron 51 casos con TB-XDR: dos casos por año en 2006 y 2007, cuatro en 2008, ocho en 2009, siete en 2010, cuatro en 2011, seis en 2012, cuatro en 2013, seis en 2014, tres en 2015 y cinco en 2016, con un promedio de cinco casos por año.

De las 33 entidades territoriales del país y durante el periodo de estudio, en 8 (24 %), se registró, al menos, un caso de TB-XDR. El 90 % de los casos se registró en los departamentos de Antioquia [n=19 (37 %)], Valle del Cauca [n=21 (41%)] y Atlántico [ n=6 (12 %)]. En el cuadro 1, se presentan las características sociodemográficas y clínicas de los casos con TB-XDR.

**Cuadro 1:** Descripción de variables sociodemográficas y clínicas en casos de TB-XDR, Colombia, 2006 a 2016

Variable	Número de casos	Porcentaje
	(N=51)	
	
		
Procedencia		
Valle del Cauca	21	41
Antioquia	19	37
Atlántico	6	12
Risaralda	1	2
Norte de Santander	1	2
La Guajira	1	2
Cauca	1	2
Arauca	1	2
Sexo		
Hombre	28	55
Mujer	23	45
Edad (años)		
Menor de 5	1	2
15 a 19	3	6
20 a 24	2	4
25 a 29	8	16
30 a 34	8	16
35 a 39	2	4
40 a 44	8	16
45 a 49	6	12
50 a 54	3	6
55 a 59	5	10
60 a 64	2	4
Mas de 65	2	4
Sin dato	1	2
Régimen de afiliación		
Contributivo	17	33
Subsidiado	32	63
No afiliado	1	2
Sin dato	1	2
Forma de tuberculosis		
Pulmonar	46	90
Extrapulmonar	3	6
Sin dato	2	4
Coinfección tuberculosis-HIV		
Sí	3	6
No	19	37
Sin dato	29	57
Coinfección	3/22	14
Resistencia a fármacos de segunda línea		
Kanamicina	37	74
Amikacina	43	86
Capreomicina	31	62
Ofloxacina	45	90
Levofloxacina	8	16
Moxifloxacina	9	18
Etionamida	10	20
Resultado		
Vivo	19	37
Muerto	29	57
Sin dato	3	6

Hubo 28 (55 %) hombres y 23 (45 %) mujeres, cuyas edades oscilaron entre los 5 y los 81 años, con una mediana de 40,28. De 2006 a 2014, no se registraron menores de 15 años y, en 2015, se identificó el primer caso en un menor de cinco años con una forma meníngea, procedente del departamento de Valle del Cauca, el cual falleció.

El régimen de afiliación a salud fue el subsidiado en 32 (63 %) casos, seguido por el contributivo en 17 (33 %).

La tuberculosis fue pulmonar en 46 (90 %) casos y extrapulmonar en 3 (6 %): pleural, meníngea y miliar; los pacientes con estas dos últimas formas, fallecieron.

El registro del resultado de la prueba para HIV solo se obtuvo en 22 (43 %) casos, de los cuales, tres (14 %) fueron positivos.

En los 51 casos, se encontró resistencia contra fármacos de segunda línea, en porcentajes de mayor a menor, así: entre las quinolonas, a la ofloxacina en 45 (90 %) casos y a la moxifloxacina en 9 (18 %), y entre los antibióticos inyectables, a la amikacina en 43 (86%) casos y a la kanamicina en 37 (74 %).

Entre los 51 casos con este tipo de resistencia en el país durante el periodo de estudio, 29 (57 %) pacientes fallecieron por la enfermedad.

## Discusión

En el 2006, en el Morbidity and Mortality Weekly Report (MMWR), los Centers for Disease Control and Prevention (CDC) y la Organización Mundial de la Saud (OMS) publicaron el primer artículo en el que se declara la emergencia sanitaria por tuberculosis resistente y en el cual se utilizó la sigla TB-XDR, debido a que encontraron que, de 3.520 aislamientos de TB-MDR, 347 (10 %) presentaban también la resistencia a la isoniacida y la rifampicina, y resistencia, al menos, a una quinolona y un inyectable ^3^.

En ese mismo año, en Colombia, se empezó a identificar algunos casos mediante estudios puntuales en zonas con alta prevalencia de farmacorresistencia. Posteriormente, en el 2008, el Laboratorio Nacional de Referencia del Instituto Nacional de Salud estandarizó las pruebas de sensibilidad a fármacos de segunda línea, con el fin de implementar la vigilancia rutinaria de los casos resistentes en el país, y confirmar aquellos que previamente se habían identificado. Por lo anterior, desde el 2010 se registra un mayor número de casos, haciendo que el comportamiento oscile en los diez años analizados.

Los departamentos donde más se registra este tipo de resistencia, son aquellos donde históricamente se ha presentado el mayor número de casos de tuberculosis y farmacorresistencia del país; estos son Antioquia, Valle y Atlántico ^5^^,^^8^. Sin embargo, cada año se van sumando casos de otras entidades territoriales, lo cual puede deberse a procesos migratorios internos de la población, deficiencias en las medidas básicas de aislamiento y control de infecciones, debilidades en los estudios de contactos de los casos diagnosticados y las actividades de seguimiento al tratamiento por parte de los programas de tuberculosis, así como a la presencia de factores determinantes sociales, como la pobreza y el hacinamiento, entre otros ^9^^,^^10^.

La tuberculosis es una enfermedad que afecta la calidad de vida de las personas, alterando de alguna manera las capacidades físicas y sicológicas para realizar actividades diarias como trabajar o estudiar. Este aspecto es importante si se tiene en cuenta que la mayoría de los casos de TB-XDR se presenta en población joven económicamente activa, situación que puede repercutir en la economía de las familias que, además de enfrentar la enfermedad, deben asumir la disminución de sus ingresos debido a la incapacidad de la persona afectada, al desplazamiento hasta la entidad de salud, a la ingestión diaria de medicamentos y a la regularidad de los controles médicos ^10^^,^^11^.

Durante el período de estudio, se presentó un caso de TB-XDR meníngea en un menor de cinco años, que concluyó con su deceso. Esta forma de la enfermedad se asocia con altas tasas de mortalidad (15 a 40 %) y secuelas neurológicas en 20 a 25 % de los supervivientes; además, puede ser un indicador de las bajas coberturas de la vacunación con BCG. Este caso es reflejo de que el país enfrenta una realidad que sigue subestimándose y que es preocupante debido a que la resistencia en niños se debe principalmente a la transmisión de cepas resistentes de los adultos; además, es un indicador de la transmisibilidad de la enfermedad y de las fallas en los programas de tuberculosis en todos los niveles de atención ^12^^-^^15^.

En cuanto al régimen de afiliación en salud, los datos son similares a lo observado en la notificación de este evento en el país ^8^.

La forma pulmonar es la más frecuente, similar a lo que sucede con esta enfermedad en general. Sin embargo, la aparición de un caso de TB-XDR debería generar una alerta en salud pública, debido a la probabilidad de transmisión de la micobacteria por vía aérea, y requeriría que las acciones programáticas se encaminen a priorizar y reforzar el estudio de todos los contactos de la persona afectada, las medidas de control de las infecciones en todos los ámbitos, y la administración oportuna y adecuada del esquema de tratamiento ^8^^,^^9^.

La prueba para HIV se registró en menos del 50 % de los casos y se observó una coinfección del 13,6 %, similar a lo reportado a nivel nacional (11,1 %) para 2017. Es de resaltar que la coexistencia de TB-XDR y HIV se convierte en un gran reto, debido a que los pacientes tienen una mayor probabilidad de presentar reacciones adversas por el número de medicamentos que deben tomar diariamente, lo cual complica la observancia del tratamiento y puede aumentar las tasas de mortalidad ^8^^,^^16^.

En cuanto a la resistencia a los fármacos de segunda línea, el mayor porcentaje de resistencia contra las quinolonas le correspondió a la ofloxacina, con 45 (90 %) casos. Esto se debe a que, hasta 2015, solo se evaluaba esta quinolona en las pruebas de sensibilidad realizadas en el país y, a partir de 2016, se incluyeron la moxifloxacina y la levofloxacina. Con respecto a los antibióticos inyectables, el mayor porcentaje de resistencia se presentó contra la amikacina, seguida de la kanamicina. En Colombia, no se encontraron estudios similares que permitan comparar los presentes resultados. No obstante, Llerena, et al., encontraron una mayor resistencia contra kanamicina y ofloxacina ^6^.

En Marruecos, en un estudio de 524 pacientes con TB-XDR, se encontró que el 95 % de ellos tenía tuberculosis resistente a la ofloxacina, el 81 %, a la kanamicina, y el 95 %, a la amikacina, similar a lo encontrado en el presente estudio ^17^.

En 2017, Sunny comparó unos casos de TB-MDR con otros de TB-XDR, y encontró una mortalidad de 16 a 25 % en los primeros, comparada con una de 50 a 60 % en los segundos, resultados similares a los obtenidos en este estudio ^18^.

Entre las limitaciones de este trabajo, se encontró que la base de datos de las pruebas de sensibilidad del Laboratorio Nacional de Referencia presentó un gran porcentaje de registros sin información sobre las variables: realización de la prueba para HIV, otros factores de riesgo o comorbilidades e historia de tratamiento, para determinar si fueron casos primarios o adquiridos.

Esto se debe a que, en el proceso de solicitud de la prueba, por lo general, el médico tratante no conoce el formato y, muchas veces, la información se completa porque el personal del Laboratorio de Salud Pública que la remite al Laboratorio Nacional de Referencia, la revisa; sin embargo, no existe un sistema nacional que permita obtener datos completos y confiables.

La información sobre antecedentes de tuberculosis y contactos del caso, permite saber si la resistencia es adquirida (debido a la inadecuada administración de medicamentos) o primaria (debido al contagio de la enfermedad con una persona cercana). Esto se documentó en los estudios realizados en el Perú, en el Hospital Nacional Hipólito Unanue y en el Hospital PNP ‘Luis N. Sáenz’, donde el 98 y el 78 % de los casos con TB-XDR, respectivamente, tenían antecedentes previos de tuberculosis; además, en el primero, se observó que el 76 % de las personas estudiadas estuvieron en contacto con pacientes con tuberculosis, en su mayoría (87 %), con formas resistentes ^19^^,^^20^.

La TB-XDR se presentó principalmente en las formas pulmonares, las cuales aumentan la probabilidad de transmisión en la comunidad, lo cual se refleja en la aparición de resistencia en menores de 15 años. La población más afectada es la económicamente activa, con una alta tasa de mortalidad. Por tal razón, el Programa Nacional de Control de Tuberculosis debe generar estrategias para controlar su diseminación, considerando el diagnóstico oportuno de la resistencia y el tratamiento adecuado, así como un manejo integral en salud y a nivel social y, fortalecer los estudios de los contactos de estos casos en el país.

La ‘Estrategia fin a la tuberculosis’ promulgada por la OPS/OMS en 2015 y adoptada por Colombia en el ‘Plan estratégico hacia el fin de la tuberculosis, Colombia 2016-2025’, se refiere a la atención centrada en el paciente y el acceso universal a las pruebas rápidas y con calidad ^21^^,^^22^. Es fundamental que el país cuente con un algoritmo diagnóstico que garantice esto en todos los casos y que, de forma programática, se garantice la vigilancia de los fármacos de segunda línea en los casos con tuberculosis farmacorresistente.

Los datos presentados evidencian que esta forma de la enfermedad afecta a niños y adultos jóvenes, acortando su expectativa de vida, en especial, al mantener la cadena de transmisión. Se requiere una directriz de manejo y seguimiento integral de los casos, acorde con las necesidades de las personas afectadas.
